# A Controversial Medicolegal Issue: Timing the Onset of Perinatal Hypoxic-Ischemic Brain Injury

**DOI:** 10.1155/2017/6024959

**Published:** 2017-08-13

**Authors:** Vittorio Fineschi, Rocco Valerio Viola, Raffaele La Russa, Alessandro Santurro, Paola Frati

**Affiliations:** ^1^Department of Anatomical, Histological, Forensic and Orthopaedic Sciences, Sapienza University of Rome, Viale Regina Elena 336, 00185 Rome, Italy; ^2^IRCCS Neuromed, Via Atinense 18, 86077 Pozzilli, Italy

## Abstract

Perinatal hypoxic-ischemic brain injury, as a result of chronic, subacute, and acute insults, represents the pathological consequence of fetal distress and birth or perinatal asphyxia, that is, “nonreassuring fetal status.” Hypoxic-ischemic injury (HII) is typically characterized by an early phase of damage, followed by a delayed inflammatory local response, in an apoptosis-necrosis continuum. In the early phase, the cytotoxic edema and eventual acute lysis take place; with reperfusion, additional damage should be assigned to excitotoxicity and oxidative stress. Finally, a later phase involves all the inflammatory activity and long-term neural tissue repairing and remodeling. In this model mechanism, loss of mitochondrial function is supposed to be the hallmark of secondary injury progression, and autophagy which is lysosome-mediated play a role in enhancing brain injury. Early-induced molecules driven by hypoxia, as chaperonins HSPs and ORP150, besides common markers for inflammatory responses, have predictive value in timing the onset of neonatal HII; on the other hand, clinical biomarkers for HII diagnosis, as CK-BB, LDH, S-100beta, and NSE, could be useful to predict outcomes.

## 1. Introduction

Defining the field of interest of this review is not that easy as it could seem. Dealing with perinatal hypoxic-ischemic brain injury, in fact, it is possible to find in literature quite different definitions and diagnostic categories. When we use the term of hypoxic-ischemic injury (HII) to the developing brain, we are referring to what already happened to a baby we probably have followed from at least labor and delivery until the onset of a patent encephalopathy. Otherwise, clinicians and especially gynecologists and obstetrics among them facing a pregnant woman with trouble in the meanwhile of delivery would generally talk about fetal distress and birth or perinatal asphyxia [1]. Furthermore, in the last few years, they prefer to talk about “nonreassuring fetal status” instead of fetal distress as it is hard to establish the effective fetal conditions before evaluating the baby at birth and along his first days of life [2–4]. HII represents the pathological consequence of the aforementioned conditions. It is hard to establish an exact correspondence between the two because often no neonatal trouble follows a clearly established fetal distress and a difficult labor or delivery [5].

These pathological conditions can be divided into 3 main subtypes depending on the timing of onset. We should differentiate, then, among chronic, subacute, and acute insults. The first is usually easy to recognize as there is an evident perturbation of normal intrauterine development during the second or early third trimester of gestation; usually, there are clear correlates at placental pathology, too [6]. These cases most of the times come to an intrauterine fetal death or a stillbirth; even if labor and delivery are prolonged or difficult, this fact represents a consequence rather than a cause of the newborn pathological development. Otherwise, subacute and acute forms happen in the perinatal interval which could start even 48 hours before birth; they are not easy to distinguish from each other as there is a lack of clinical and instrumental parameters for clearly identifying a prenatal injury. Moreover, it should be taken into account that a subacute condition may evolve into an acute one; the main difference, in fact, is the quickness which is more evident in the acute form and leads to a faster evolution from the initial insult to the lesions and clinical correlates.

Outcomes of subacute and acute fetal distress range from intrauterine death, stillbirth, and neonatal death to neonatal encephalopathy. This is a general term preferred to the more specific hypoxic-ischemic encephalopathy (HIE) when it is still undetermined whether the cause has been a perinatal HII or not [7]. By the way, a universally agreed definition of both these terms is still lacking [8].

The problem we want to stress with the present review is that in the real setting, the abovementioned conditions overlap so that it is hard to assess an etiological diagnosis to a given case. Even chronic fetal injury may pass unnoticed during pregnancy, and its pathological correlates may be confused with subacute and acute events. Another factor which complicates this picture is the gestational age of the fetus because it is well known that prematurity by itself leads to additional risks and vulnerability in the early postpartum period.

From the clinical point of view, it could seem not that important to address every consequence to the specific cause and relative onset, but it is paramount in the forensic and medicolegal setting where the judgment about the medical liability should be set upon the ascertainment of the causal relationships, both in civil and penal trials.

### 1.1. Epidemiology

In the last few years, intrapartum-related neonatal deaths have been assessed at the fifth place in the most common causes of death chart among children less than 5 years old [9]. This makes a worldwide estimate of more than 800,000 deaths per year. Considering all perinatal fatal outcomes, these cases represent about a quarter of total 3 million deaths and almost half of 2.6 million third trimester stillbirths [10].

Dealing with different reports and meta-analysis regarding full-term births [11], perinatal asphyxia has an incidence of 1–6 per 1000 live births with a mortality rate up to 20%. About one-half of survivors suffer from neuropsychological sequelae of immediate or delayed onset [12] while 25% show major neurological impairments.

If considering neonatal encephalopathy, it is estimated that from 1 to 3 per 1000 live births, a half of them were accountable for a hypoxic-ischemic causation [13].

In these data, there are significative discrepancies between high-income countries, developing, and low-income ones; in the first ones, survival rate is definitely better while the latter ones show an incidence of neonatal encephalopathy lower than expected. This is obviously due to the underestimation related to the shorter overall survival time which does not allow encephalopathy to take place. Moreover, it can be estimated as a kind of evidence for intrapartum hypoxia-ischemia in the 30% or 60% of cases, respectively, in developed and developing countries.

### 1.2. Role of Perinatal Hypoxia

In the case of chronic intrauterine insult, it is possible to identify clear etiopathogenic factors, such as placental insufficiency, chorioamnionitis, genetic anomalies, or toxic exposure. Otherwise, when brain injury occurs in an acute or subacute manner, lots of different factors may have played a role.

Birth asphyxia has been defined as “a condition of impaired blood gas exchange, leading, if it persists, to progressive hypoxemia and hypercapnia.” Unfortunately, this statement does not provide any precise clinical parameter to affirm such a diagnosis in a given case or in a study cohort.

Main clinical events associated with perinatal asphyxia can be summarized as the following: placental abruption, meconium staining, labor dystocia (abnormal uterine contractions, obstructed labor), cord knots, cord prolapse, and uterine rupture. A significant problem is that sometimes clinicians just ascertain “the failure of the neonate to successfully begin breathing,” not surprisingly the WHO in its “practical guide to newborn resuscitation” preferred to choose the latter as the only definition provided [14]. Briefly, we have to face a complex scenario where causes and consequences, as clinical correlates of perinatal asphyxia, confuse with each other.

In some recently published papers, the authors seem to treat “birth asphyxia” and “perinatal hypoxia-ischemia” as perfect synonyms [15], but this cannot simplify the problem by itself. As a matter of fact, although the most objective assessment of the presence of intrapartum hypoxia-ischemia is metabolic acidosis in umbilical arterial blood at the time of birth [16], not all the authors refer to the same cut-off values or strictly measure these parameters in their studies. Basic controversies exist also regarding the definition of neonatal encephalopathy (NE) and hypoxic-ischemic encephalopathy (HIE) [17].

All these facts explain partially why authors disagree so much about incidence of intrapartum causes in neonatal brain injury: some of them affirm a minor role [18–20], while others still consider them surely predominant [21, 22], at least when major congenital malformation and chromosomal disorders are excluded [23, 24].

Another reason for disagreement is that nowadays, it is well established that cerebral palsy in children is linked to an intrapartum event only in the 10–20% of cases [25]; it is necessary to keep in mind that often cerebral palsy does not have any pathological presentation in the first weeks of life. Indeed, encephalopathic babies at birth and children affected by cerebral palsy are not two identical groups.

### 1.3. The Medicolegal Matter of Interest

The reported epidemiological data underline that HIE is not only a public health concern. In the last two decades, medicolegal claims arisen from perinatal brain injury have been always growing. Recently birth-related events have been estimated to account for about a half of the cases dealt with by the National Health Service Litigation Authority in the UK. If it is well known as medical malpractice litigation in the developed world that has reached huge proportions, an important place should be assigned to neonatal brain injury compensation. According to the report of a consortium of insurance companies in the USA, this kind of damage is at the top of the list of pediatric claims (from 1985 to 2008) with an average indemnity of about 500,000 dollars [26].

The present system, especially in the civil courts, is outcome-based, and this fact consists of a guilt prejudice upon physicians. This depends notably on the lack of clear and univocal criteria but also on the unavailability of those proofs and tests which allow reaching the causal correlation on the basis of evidence. This uncertainty results in unpredictable judicial trials with similar cases often judged in the opposite way: conviction and discharge verdicts, thus, are based sometimes on nonobjective medicolegal and forensic evaluation, especially when some elements are ignored or neglected.

Medicolegal evaluation is certainly much more oriented when dealing with a fatal case so that a judicial autopsy has been disposed. In literature, it is largely represented how full postmortem examination, including a complete autopsy, may provide elements of paramount importance from a quantitative as long as a qualitative point of view [27, 28]. Nevertheless, autopsy alone cannot provide all the solutions to the correct decision.

Timing of injury is definitely the key element in the legal arena as the defense will always try to demonstrate that injury occurred in a different moment rather than the strict intrapartum period; the confutation of causality relationship, in fact, will be overwhelming on claims about eventually debatable physicians' misconduct [29]. By the way, when cases are particularly delicate, especially in civil courts, it is necessary to finely distinguish concomitant causes between human and natural ones [30], then natural causes between preventable and unpreventable ones; at the same time, an explication of the relative weight of each of them should be given. Easy to see how the timing of injury and consequent impairment is an essential question, not limited to the simple assessment whether it is an antepartum or intrapartum onset but regarding the promptness of procedures performed by all the health assistance personnel too.

## 2. Pathobiology of Hypoxic-Ischemic Brain Injury

### 2.1. Pathophysiology

As it is not easy to identify the exact etiological factor related to HII to the neonatal brain, in the same manner, there is still uncertainty about all processes leading to the well-described neuropathological features reviewed below. The first event in the pathophysiology of this condition is the instauration of central fetal hypoxemia, since the impairment in feto-placental hemodynamics and blood gas exchange is established [31]. Quickly hypoxia is followed by ischemia; this is the reason why we talk about a “hypoxic-ischemic” injury and both components should always be considered together in the subsequent lesions. Thus, in addition to reduced oxygen, also lessening of glucose supply and increasing of carbon dioxide are involved in the brain damage leading to the complex metabolic impairment which is characteristic of HII [32].

It has been demonstrated that ischemia is induced by the combination of myocardial dysfunctions and the loss of cerebral self-regulation of blood flow. Intracranial hypotension and hypoperfusion worsen tissue anoxia and reduce metabolic substrate supply. By the way, the critical characteristic of HII in the perinatal period is the intrinsic vulnerability of the immature brain [33, 34]. This depends on the high energetic need of neurons, specific interneuronal connections [35], and immaturity of oligodendrocyte lineage [36, 37].

The early phase of damage involves vascular mechanisms too. From one side, vasoconstriction takes place and, above all, hypotension may lead to vascular collapse; from the other, the endothelial damage at the microcirculation causes a marked opening of the blood-brain barrier. When the strictly selective permeability of this district is compromised, toxic agents are licensed to pass and worsen brain damage.

The early phase of damage, which lasts for the first 24–48 hours from the initial insult, is followed by a delayed injury [38]. This is mainly driven by the inflammatory local response [39, 40]. Focusing on the further development of the central nervous system, the perinatal HII can be interpreted as the initial *noxa* of a subsequent altered neural migration and differentiation due to hypoxic-induced angiogenesis and vascular activation [41, 42].

Finally, when considering the complex pathophysiology of HIE, one should remember that general conditions of the newborn, which usually complicate perinatal asphyxia (glucose, electrolytes, and ammonium imbalance), may play a role in worsening brain damage and precipitating mid and long-term prognosis [43].

### 2.2. Molecular Pathways

Neurons affected by a hypoxic-ischemic insult undergo usual effects as seen in general cytopathology. The failure of oxidative energetic metabolism in providing a sufficient level of ATP determines the loss of intracellular homeostasis; the Na^+^/K^+^ pump function ceases and the consequent osmotic and electrochemical gradient produces the cell swelling. Depending on the intensity and prolongation of the insult, this process can reach an acute cell lysis.

By the way, in most cases of brain injury, there is a network of cross-talking cellular processes that results in the combination of the 2 main cellular deaths: necrosis and apoptosis. Apoptosis plays an important role in brain development as immature cells are likely more prone to apoptosis even in physiological conditions [44]. When a hypoxic-ischemic injury occurs, apoptosis and necrosis overlap so much that nowadays it is common to talk about an apoptosis-necrosis continuum in a given injured brain area [45, 46].

This fact can be explained describing a typical response to HII in the neural tissue which actually is common also to other kinds of *noxa*; it is known as “excitotoxicity” [47] and shows how the accumulation of excitatory amino acids (EEAs) in the extracellular medium promotes neuronal death [48]. The mechanisms initially derived by in vitro experiments and confirmed by *in vivo* models are like a vicious cycle in which the central role is held by NMDA receptors [49]. These, in fact, are activated by extracellular glutamate (the main excitatory neurotransmitter of CNS) and drive a self-perpetuating influx of Ca2^+^ involving the Na^+^ channels [50]. This triggers all the enzymatic Ca-dependent cascades which lead both to apoptosis and necrosis [51].

It could be that the pivotal role in determining a more oriented progression towards necrosis rather than apoptosis is played by the specific mitochondrial dysfunction [52]. This damage is being deeply investigated in the field of brain injury because the loss of mitochondrial function is supposed to be the hallmark of secondary injury progression [53].

All these mechanisms have been reviewed recently [54]. It is well depicted that there are at least three chronologically distinct stages: in the early phase, the cytotoxic edema and eventual acute lysis take place; with reperfusion, additional damage should be assigned to excitotoxicity and oxidative stress; finally, the later phase involves all the inflammatory activity and long-term neural tissue repairing and remodeling. This confirms that time-dependent representation of neuropathological findings, as in more details below, has a biological and molecular basis because only a few neurons die during the actual ischemic event while a delayed cell death continues along a latent phase.

Nevertheless, this chronological distribution alone cannot provide a reliable paradigm to answer the question if a given HII started during labor and delivery or it is linked to a previous insult.

### 2.3. Novel Insights into Injury Mechanisms

Besides apoptosis and necrosis, a more recently discovered kind of cellular death is being investigated in relation to neonatal brain injury, that is, autophagy. Autophagy is a controlled lysosome-mediated cellular function to eliminate damaged or aged organelles and to maintain cell survival under multiple stresses; unfortunately, under certain conditions, this homeostatic process results in cell death [55]. Historically, it seemed at least ambiguous if this peculiar adaptative response could play an effective protective function in the field of brain HII. In literature, it was possible to find opposite conclusions to this crucial question: some authors, applying on experimental pharmacological tests, argued that autophagy actively contrasted lesion progression, limiting the extension of necrosis [56]; at the opposite side, some authors, using knockout rats, concluded that this process was deleterious [57].

We believe that the recent research by Ginet et al. [58] provides a univocal and reliable answer. They combined an *in vitro* and *in vivo* approach in the same experiment and showed clearly from both sides the pathological role of autophagy while a hypoxic-ischemic insult acts: autophagy adds itself to the apoptosis-necrosis continuum and enhances brain injury. It is remarkable how they obtained *in vivo* proofs implementing the technique of inducing specific gene downregulation with targeted lentiviral vectors.

With similar experimental strategies, other molecules have been linked to central mechanisms in this kind of neuronal lesions. Recently, the potential role of the oxidative product hydrogen sulfide (H2S) has been highlighted [59]: according to the work by Lechpammer et al. [60], it connects the injury-related activation of the enzyme cystathionine-beta-synthase with the mTOR-dependent pathway. The authors, in fact, found a neuroprotective potential with rapamycin, the well-known prototypical inhibitor of mTOR.

Fascinating insights into HII pathogenesis come from epigenetic studies, even if they were originally set for cancer and tumor cells [61]. Researchers in this field started from a deep investigation of gene expression in the developing brain, focusing on the checkpoints in cellular and tissue differentiation. Then they looked for molecular links between already known injury triggers and intermediate or late processes [62]. The epigenetic dysregulation leading to the main cellular enzymatic activity involved in all mechanisms seen above is being depicted [63]. Epigenetics can explain also which way specific conditions interact and worsen HII. It happens, for example, when fetal antenatal stress and consequent epigenetic repression of GR (glucocorticoid receptor) brain expression abrogate the neuroprotective action of corticosteroids [64, 65]. According to the state of art among miRNA, which are the most investigated protein expression modulators, the ones that have been related to hypoxic-ischemic brain injury are miRNA 210 [66], a well-known hypoxia upregulated mediator [67], miRNA 128 [68], and, above all, miRNA 9.

All these experimental evidences are still far away to be applicable in the clinical setting; they are insufficient to clarify all the aspects of pathobiology in HII of the neonatal brain too. Nevertheless, we believe that scrutinizing and mapping also in a time-sensitive manner, the spectrum of miRNA expression in damaged and perilesional tissues could provide very precise diagnostic tools if matched with advanced microscopy and molecular biology.

## 3. Assessment of Timing of the Onset

### 3.1. Clinical Approach

About clinical presentation of HIE, no certain time relationship can be established with timing of injury. It has been observed that latency for first observable seizures can range from minutes to a few days from birth even when a clear asphyxial event occurred [69]. These ranges might preclude assignment of an injury to the time of labor [70].

Some authors reviewed the literature for identifying a reliable timer, and they assumed that persistent fetal bradycardia could work; by the way, it was only obtained from primate experimental models which have been largely criticized because of poor similarity to the real clinical setting. Some other parameters have been tested such as lymphocytosis and thrombocytopenia in the neonate blood by retrospective medical chart review. Conclusions admitted that even if lymphocytosis may be reliable, it refers only to a strict time window between initial insult and blood sampling; furthermore, it is applicable only in definite conditions and lots of confounding factors exist.

Another problem is the late-referral pregnant women with labor troubles; if there is no previous recent clinical observation, even an impressive event like fetal movement, stopping may become not valuable.

Anyway in the clinical setting defining the time of the onset is not of great importance in itself as neonatologists are more interested in quickly assigning a prognosis and send the newborn to the most proper therapeutic strategy. Nowadays, in fact, some new resources are available in this field [71] and others are being developed [72], like the intravenous administration of autologous cord blood cells [73] or an erythropoietin support [74]. However, the most important one, the medical hypothermia [75, 76], is administered only in a few centers. From the other hand, certain advanced drugs are potentially dangerous and babies who will likely not beneficiate should be exempted from the risk.

### 3.2. Neuropathology

Neuropathological findings related to neonatal encephalopathies and brain damage have been reviewed more than once in literature. We suggest referring to a few of these monographs for a more detailed description of each pattern reported below [77–79]. Our aim is to focus on controversies dealing with assessing a definite etiological diagnosis and establishing the timing of onset.

This field of pediatric neuropathology, in fact, is characterized by a huge variety of aspects deriving from the complex biology of neurological damage in the developing brains. Ethical issues which reasonably limit research access to death cases join the obvious unavailability of human samples coming from patients who are still alive. Lots of evidence, then, come from experimental models of mammalian neonates in which injuries are caused almost ever by total occlusion of main cerebral arteries. One of the most referred procedure is the Rice-Vannucci which consists of unilateral common carotid artery ligation, followed by hypoxic environment exposure (pO2 = 8%) [80, 81]. Usually, such an experiment produces an “all or none” phenomenon which is clearly limiting comparing with the number of combinations of different lesions observed in real human cases.

Classical patterns have been divided into subtypes and kind of classes of frequency dealing with the gestational age of the newborn. However, we do not consider this classification useful enough for our principal intent as we are interested the most in assigning lesions to a precise chronological relationship with labor and birth. Before summarizing these aspects, it is important to remind that lots of factors besides timing of the onset contribute to the specific pathological picture. First of all, it is mandatory to consider the duration of the insult, which determines greatly but not entirely the intensity of the insult itself; the comorbidities, again, both from fetal and maternal side; the effective fetal growth with respect to gestational age; and the topographic distribution when mechanical forces are supposed to have been involved in the injury.

Considering all these facts in literature, there is a consensus in recognizing at least 3 stages in the pathological progression of developing brain damage [82]. In an “acute” phase (restricted to a time window of 8–24 hours), aspects of cellular death prevail, with hypereosinophilic neurons and nuclear pyknosis in the gray matter, while coagulative necrosis and classical axonal spheroids in the white matter. The “subacute” inflammatory phase is considered starting at least 72 hours after the first onset and is characterized by macrophage infiltration or small collections and “gliosis” (glia activation), which means microglial nodules surrounding dead neurons and mineralization of cell residues (gray matter), and reactive astrocytes (both gray and white).

In this progression, someone else [83] individuated another time window in the acute phase with appearance of neuronal karyorrhexis, which would occur between 24 and 48 hours but this is not elsewhere accepted in literature. What is undoubted, instead, is the label of chronic sequelae (weeks-months) for gross pathological appearances like cavitation or coarse spongiosis of the cortex or periventricular cysts and glial scars. A well-described late pattern is the “marbled state” of basal ganglia, which is supposed to be the effect of an altered process of reactive long-term hypermyelinization of nonaxonal fibers. The counterpart in the white matter could be recognized in the so-called “periventricular leukomalacia” (PVL), which consists of 2 major components: the results of focal necrosis of deeper white matter (cysts, scars, but most of all ventriculomegaly) and more diffuse injury with marked microgliosis and astrocytosis. This aspect has been elsewhere depicted as DWMG (diffuse white matter gliosis), characterized by hypertrophic astrocytes, capillary cell proliferation, and perivascular “globules” eventually mineralized [84].

The chronological progression proposed is clearly dependent on survival interval from injury and subsequent *in vivo* modifications. The derived assessment of the timing of the onset is based on a retrospective (a fortiori) measurement, with no intrinsic relationship with labor- or delivery- associated events. Another problem is that has been well demonstrated how the correspondent acute phase of a chronic basal ganglia and thalami-altered morphology can result just in subtle findings of apoptosis and/or necrosis which may be easily overlooked histopathologically.

Furthermore, the utilization of the aforementioned parameters implicates several limitations: the time windows are too long, being able to include both antepartum and postpartum events; there is a time “hollow” between 24 and 72 hours with no specific findings; and it is of no usefulness after a few days from delivery.

To overtake these limitations, we would need better knowledge about correlation between the kind of intrapartum insult or risk factor-associated (see above) and topographical or qualitative representation of lesions and respective pathological sequelae. By now, we can just consider that some disorders of gyration with observable cytoarchitectural disorganization refer typically to a certain gestational interval; similarly, we know that PVL, when already established at birth, is likely related to an insult occurring between the 24th and the 34th gestational week. These correlations are consistent with the well-known principle that lesional pattern in the developing brain is determined by specific neuronal susceptibility to hypoxic-ischemic injury related to structure and morphogenesis.

Another aspect, which deserves analysis and further research, is the effect of injury timing in determining the particular combination of grey and white matter. We are talking about the possible association with PVL of damage localized in the grey matter, at the subplate neurons, cortex, basal ganglia, and thalami [85].

### 3.3. Neuroimaging

Neuroimaging is gaining more and more importance for neonatologists as it allows searching for a pathological counterpart in the clinical setting. Almost all, both in research and real world, deal with MRI, as CT scans need dangerous ionizing radiations and transcranial ultrasound exams have poor sensitivity [86]. Moreover, technical advances and knowledge exploitation in performing all possible sequences and parameters have improved its diagnostic usefulness and potential application in timing the onset of injury.

Pathological patterns have been classified traditionally in 4 types on a topographical involvement basis: deeper structures, cortex, periventricular white matter, and mixed [87]. It is clear that images at MRI are not comparable with neuropathological observable patterns in term of resolution and detail characterization; from the other side, they share the same variability depending on not only intensity and prolongation of hypoxia-ischemia but most of all on developmental stage of neonatal brain and on time span from injury to exam [88]. First, we should notice that, once again, this lag time is determined by the timing of clinical presentation, rather than effective onset of brain injury. Then, it is controversial in literature when should MRI be performed on neonates when there is a clinical question about eventual brain lesions.

Besides mere recognition of abnormal patterns and support to definite diagnosis, in fact, clinicians show interest in neuroimaging as it can lead to an earlier assessment of disease and even prognosis [89, 90]. Dealing with the first issue in someone's opinion, the hypoxic injury pattern on MRI is not diagnostic for 7–14 days [91], as perinatal brain lesions are at their most visually obvious state between 1 and 2 weeks from delivery [92]. This fact could be an important limitation in those cases characterized by a shorter survival interval [93]. At the same time, the finding of diffusion restriction at DWI-MR is accounted for enabling early detection of injury, even on the first day [94]; this is the reason why some authors assert that this kind of sequence imaging is optimally obtained between 3 and 5 days from delivery [95]. In any case, diffusion-weighted parameters still lack sensitivity and may lead to underestimate the extent of lesions [96, 97], especially when deep gray matter is involved. One more method implemented to anticipate HIE detection at MRI is to combine conventional imaging acquisition and proton MR spectroscopy for anaerobic metabolism markers (increased lactate and decreased N-acetyl-aspartate) [98]. Anyway, not all the authors agree about its feasibility because of poor specificity. Lastly, diffusion tensor imaging (DTI) modifications are being mapped in relation to neonatal brain HII [99, 100] and long-term outcomes [101].

About prognosis, a specific MRI scoring system was proposed already in the 90s, but it consisted of a weighted analysis of topographically different signal abnormalities to find a correlation with neuromotor impairment during the first 12 months of life [102]. Rather than being a real prognostic tool, it helps to the interpretation of subtle findings when clear pathological patterns are not evident. This is the same for the discussed “prognostic” value of signal changes in the posterior limb of the internal capsule (PLIC) [103].

If uncertainty exists about effective time specificity of signal changes at each MRI acquisition sequence, neuroimaging exams obtained in the first weeks of life are believed effective in excluding an ancient antepartum process [70]. Usually, in fact, they are used in clinical studies as criteria suggestive of antenatal insult when revealing typical late sequelae already mentioned in the neuropathology paragraph or generalized encephalic malformations and developmental anomalies. Similarly, signs of brain swelling, cortical highlighting, loss of gray-white matter differentiation, signal abnormalities in basal ganglia, and thalami are usually intended to be suggestive of perinatal insult. However, none of these signs is completely specific, as each one of them has been associated with the evidence of placental inflammation and no asphyxial birth event [104]. Not surprisingly even when discussing the results obtained from large case series, neuroradiologists agree that imaging findings should be reviewed in the context of the clinical setting to determine the underlying etiology and time of onset in HII.

At the end, we should not forget that even in the most accurate and advanced application, false negative cases of subsequent neurological defects are still observable at MRI [105], and this fact limits significantly its overall negative predictive value. Surely, instead, neuroimaging represents the only surrogate of a pathological assessment in nonlethal cases.

## 4. New Perspectives for an Objective Evaluation

### 4.1. Limits of the Actually Available State of Art

It is not easy to delineate a proper scenario from all these evidences, usually conflicting with each other. The most believable conclusion should be that there are two apparently incompatible points of view.

From one side, clinicians' scientific societies tend to assign an intrapartum or at least partum-related etiology for neonatal brain injury and neurodevelopmental disorders in a few cases only [106]. According to this, it would be possible to define it only for contemporary assessment of acidosis at umbilical cord arterial blood sample, early onset of moderate-severe encephalopathy, spastic or dyskinetic neuromotor impairment, and absence of other possible causes [107]. At the other hand, neuropathological-oriented reviews still consider the prevalent role of birth asphyxia and peripartum onset of brain insult. Thus, they consider the criteria reported as too restrictive and essentially unfit to describe the real epidemiology.

In order to widen clinical evaluation, a few interesting diagnostic checklists have been proposed [108, 109]. They consist of nonweighted lists of indicators, based predominantly on maternal and gestational clinical history, that are largely evaluated in literature as singular risk factors for neurological impairment of childhood. Unfortunately, this kind of clinical tool is hardly decisive in a judicial setting because, in the given case, usually more than one factor coexist, attributable to opposite causation according to the checklist itself. It is even harder in the case of penal trials where the judge pretends from the forensic pathologist or the medicolegal consultant to answer based on certainty (or “almost certainty”).

At the state of knowledge, we reviewed by now in this work that it would be theoretically possible to trust in neuroimaging to assess if an insult occurred during labor and delivery or was already established during pregnancy. However, in the real world, the actual availability of the needed exams is inadequate to solve most of the cases. Moreover, expert radiologists in this high specialized and difficult field is still lacking. Obviously, as we said before, when survival interval of the newborn is short or a stillbirth occurs and an autopsy is performed, neuropathological findings strongly prevail on imaging. Nevertheless, lots of cases, especially in civil courts, are debated without any autoptic result. In other circumstances, death might happen after a considerable lag time since birth so pathological findings would be nonspecific to time the onset while MRI scans, if obtained during the first days of life, it would be more useful.

Anyway, notwithstanding the availability of exams and observations at both clinical imaging and postmortem pathology, all the parameters seen are subjective and operator-dependent. This represents a considerable limit to the exploitation of similar evidences for the burden of proof in a given trial setting.

### 4.2. New Tools for an Evidence-Based Medicolegal Assessment

Forensic pathology shows rising interest in implementing immunohistochemistry essays in order to corroborate qualitative evidences with semiquantitative parameters. Not surprisingly, then, different immunohistochemical markers have been tested for their predictive value in timing the onset of neonatal HII. First of all, common markers for inflammatory responses, such as CD68, CD45, KP1, and HAM56, react for activated microglia or macrophages; more specifically in the nervous tissue, immune stainings for glial fibrillary acidic protein are commonly used to highlight reactive astrocytes. These reactions are believed to be reliable to time the onset of injury at 1 or more days before the examination, at least; they have a comprehensible limit in still poor resolution of the lag time, and they are also nonspecific in relation to the kind of insult.

Specific apoptosis immunohistochemical essays, like TUNEL (terminal deoxynucleotidyl transferase-mediated deoxyuridine triphosphate nick end labeling), Bcl-2, and Bcl-x, have been tested to characterize hypoxic-ischemic brain lesions, but it was not made in the timing perspective [110]; the same happened with the VEGF (vascular endothelial growth factor) mapping in hypoxic central nervous tissue. More recently, instead, Riezzo et al. provided precious evidences searching for early-induced molecules driven by hypoxia [111]. They found a well-defined pattern in chaperonins HSPs and ORP150 (osteogenetic regulatory protein) over time expression; they concluded that positivity for both HSP70 and ORP150 is consistently associated with a lag time from the insult of less than 8 hours, while HSP90 turns positive only after this time span until about 48 hours. Not surprisingly, HSP70, that had already been associated with ischemic nervous tissue [112], could be a protective cellular factor from apoptosis by maintaining Ca2^+^ homeostasis [113].

In the future studies, we can hypothesize a growing deepness in molecular mapping of injured brain tissue, not only in relation to time but also to the type of injury, as we already see with the application of advanced blotting techniques [114] and quantitative RT-PCR for extracted RNA [115]. Surely, big efforts will interest microdissection and targeted miRNA and gene expression analysis.

The counterpart of objective immunohistochemical and molecular data for the nonautoptical cases could become soon the serum or cerebrospinal fluid biomarker for neonatal encephalopathy [116, 117]. At present, clinically used biomarkers to aid the diagnosis of HIE are quite general cytolytic ones like creatine kinase isoenzyme (CK-BB) and lactic dehydrogenase (LDH). Lots of others are being evaluated in the experimental setting in consideration of molecular characterization of central nervous tissue, by analogy with brain trauma research [118]. A recent review on potential biomarker proposes the implementation of GFAP and the spectrin protein breakdown products as reliable serum markers to assess brain injury. According to other authors, neuronal protein S-100betha and neuron-specific enolase (NSE) could be useful to predict outcomes in encephalopathic babies [119, 120] while the glial to neuronal biomarker ratio has been proposed as an indicator to differentiate focal and diffuse injury [121]. Notably, none of these has been studied for an etiological diagnosis or as a timer [122] but it is reasonable to think that if they became common in clinical practice, their real-time correlation with clinical features would be really useful to time the onset, especially retrospectively.
